# Data Processing Strategies to Determine Maximum Oxygen Uptake: A Systematic Scoping Review and Experimental Comparison with Guidelines for Reporting

**DOI:** 10.1007/s40279-023-01903-3

**Published:** 2023-08-21

**Authors:** Simon Nolte, Robert Rein, Oliver Jan Quittmann

**Affiliations:** 1 Institute of Movement and Neurosciences, German Sport University Cologne, Cologne, Germany; 2 Institute of Exercise Training and Sport Informatics, German Sport University Cologne, Cologne, Germany

## Abstract

**Background:**

Gas exchange data from maximum oxygen uptake ($\dot{V}{\text{O}}_{2\max }$) testing typically require post-processing. Different processing strategies may lead to varying $\dot{V}{\text{O}}_{2\max }$ values affecting their interpretation. However, the exact processing strategies used in the literature have yet to be systematically investigated. Previous research investigated differences across methods at the group level only.

**Methods:**

Out of a random sample, we investigated 242 recently published articles that measured $\dot{V}{\text{O}}_{2\max }$ during ramp tests. Reported data processing methods and their rationale were extracted. We compared the most common processing strategies on a data set of 72 standardized exercise tests in trained athletes.

**Results:**

Half of the included studies did not report their data processing strategy and almost all articles failed to provide a rationale for the particular strategy chosen. Most studies use binned time averages to determine $\dot{V}{\text{O}}_{2\max }$, with a minority using moving time or moving breath averages. The processing strategies found in the literature can lead to mean differences in $\dot{V}{\text{O}}_{2\max }$ of more than 5% (range 0–7%) with considerable variation at the individual level.

**Conclusions:**

We advise researchers to change their processing strategy and use moving averages or digital filters instead of binned averages. Researchers should report their data processing strategy used to determine $\dot{V}{\text{O}}_{2\max }$. We provide a reporting checklist of seven items that can function as a template for reporting.

**Supplementary Information:**

The online version contains supplementary material available at 10.1007/s40279-023-01903-3.

## Key Points

**Table:** 

Despite calls to use moving averages or digital filters, binned averages remain the most common data processing strategy to determine maximum oxygen uptake. The use of binned averages is not advisable. We recommend using digital filters or, if that is not possible, a 30-s moving average.
Different processing strategies lead to varying maximum oxygen uptake values at the mean as well as at the individual level.
Researchers are advised to report their processing method in detail, or preferably share their raw oxygen uptake data and analysis code.

## Introduction

The maximum oxygen uptake ($\dot{V}{\text{O}}_{2\max }$) is one of the most commonly assessed physiological parameters in sports and exercise science [1]. Maximum oxygen uptake highly corresponds with endurance performance in heterogeneous groups [2–4] and can be regarded as one of the most relevant physiological predictors of endurance performance [1], though measures such as critical power may be better predictors, especially in homogeneous groups [5]. Accordingly, many exercise physiologists and clinical professionals use $\dot{V}{\text{O}}_{2\max }$ as a criterion measure of endurance exercise performance as well as cardiorespiratory and muscular endurance fitness/health. Changes in $\dot{V}{\text{O}}_{2\max }$ are then interpreted to reflect changes in these factors and capacities.

Researchers predominantly measure $\dot{V}{\text{O}}_{2\max }$ during exercise tests to exhaustion. The measured gas exchange data are inherently noisy as both the biological variability of breathing patterns (further complicated by irregular breaths, coughs, and swallowing) and the measurement error can result in large fluctuations of raw oxygen uptake data on a breath-by-breath basis. Therefore, the raw data require some form of processing to obtain data that better reveal the underlying system’s physiology and cellular biochemistry. However, different data processing strategies influence measured parameters of gas exchange [6, 7]. Consequently, the same oxygen uptake data generated during an exercise test may result in varying outcomes when processed differently [8]. This can have serious implications in practice [9].

As previously mentioned, an optimal processing strategy should ideally separate extraneous biological and measurement noise from the “true” physiological and biochemical determinants of pulmonary oxygen uptake. Most of the variability in measured oxygen uptake data stems from variability in breathing frequency and tidal volume [10]. Assuming that the measured data are a combination of different oscillating signals (e.g., ventilation and muscle metabolism) and measurement error, a natural approach would be to perform frequency-based filtering or at least averaging procedures that are based on the time characteristics of the physiological signals [11].

Whether an observed peak in oxygen uptake corresponds to the true maximum has been extensively discussed in previous research [12–16]. To identify a true maximum, researchers commonly evaluate a set of parameters measured during ramp tests—the ‘$\dot{V}{\text{O}}_{2\max }$ criteria’ [13]. We will not distinguish between peak and maximum oxygen uptake in this article, as the criteria for $\dot{V}{\text{O}}_{2\max }$ (e.g., the primary criterion of a plateau in oxygen uptake or the secondary criterion of the maximum respiratory quotient) do heavily rely on the data processing strategy used [8]. We did not consider any secondary criteria as their validity is questionable [14]. Thus, we define for the current purposes $\dot{V}{\text{O}}_{2\max }$ as the maximum oxygen uptake measured during an appropriate exercise test (i.e., an exercise test to exhaustion with a duration ≤ 20 min) regardless of any $\dot{V}{\text{O}}_{2\max }$ criteria.

Researchers have proposed a variety of calculation intervals and computational methods to process oxygen uptake data [11, 13, 17–19] and calls to standardize processing strategies are frequent [9, 10, 20, 21]. In light of the influence on outcome variables, many articles highlighted the need to report processing strategies in research [8–10, 21]. Midgley et al. [22] were the first to evaluate reported data processing strategies for breath-by-breath analyses in selected journals. They found that all studies reported the use of binned time averaging, with only 1 in 117 using a moving time and a moving breath average, respectively. One third of the studies did not describe their processing method at all.

Robergs et al. [11] argued that to investigate the current state of data processing strategies, two possible approaches are *“(i) a summary of published research, and (ii) a survey circulated *via* the Internet to as many exercise physiologists as possible”*. They chose the latter approach with a total of 75 respondents, who reported a large variety of data processing strategies. Most researchers reported the use of binned time averages over 30 or 60 s. Surprisingly, about half of the respondents admitted that their data processing strategy was chosen based on subjective factors as opposed to objective criteria [11]. While historically data processing from oxygen uptake data was limited by methodological and testing constraints, these limitations should not be present in the current research because of the exclusion of research prior to 2017 (see Methods). The present work therefore aims to investigate to what extent reporting and processing practices have followed the recommendations put forth by Midgley et al. [22] and Robergs et al. [11].

Selected data processing strategies have been extensively compared in the literature. Because of the absence of a systematic mapping of current practices, these studies lacked the reasoning on which strategies to compare. Many studies compared different averaging intervals, but not averaging types (e.g., moving breath vs binned time) [8, 22]. Martin-Rincon et al. [23] provided formulas for comparing data processing strategies by investigating a data set of sedentary individuals and recreational athletes, using two different metabolic carts. Therefore, in their work, motivation and measurement devices may have interacted with the influence of processing strategies. Most comparisons only report mean differences between strategies [8, 22]. No research has yet compared a variety of systematically derived strategies among a group of trained individuals using a standardized measurement set-up.

Differences in estimated $\dot{V}{\text{O}}_{2\max }$ values due to variations in data processing can have serious implications in practice. For example, the assessment of longitudinal data from athletes who participated in diagnostics using differing data processing approaches becomes problematic. The same applies to the pooling of results across studies in meta-analyses [23]. Data processing strategies directly affect the estimate of a plateau in oxygen uptake, the primary criterion for $\dot{V}{\text{O}}_{2\max }$ [8]. Crucially, in situations where individuals are classified by their $\dot{V}{\text{O}}_{2\max }$—for example, when describing the training status of a study population [24, 25] or evaluating patients for a heart transplantation [26]—differing processing strategies can lead to misclassifications [9]. As such, data processing strategies may magnify existing biases in patient and athlete evaluations [27, 28].

Despite the relevance of choosing the right processing strategy for $\dot{V}{\text{O}}_{2\max }$ determination, it is currently unclear which strategies are actively used in the recent literature and how they compare against each other on a standardized set of oxygen uptake data. This paper aims to review the usage and reporting of different data processing strategies in the scientific literature and investigates their influence on $\dot{V}{\text{O}}_{2\max }$. The results will help to compare $\dot{V}{\text{O}}_{2\max }$ data derived from different processing methods among studies and in individuals. The review allows for the assessment of the implementation of data processing routines and to identify problematic reporting strategies. The results build a basis for providing recommendations for the reporting of data processing strategies to determine $\dot{V}{\text{O}}_{2\max }$.

## Methods

The present work was preregistered before the project start with the Open Science Framework [29], following the ‘Inclusive Systematic Review Registration Form’ [30]. Any deviations from the preregistration are indicated in a ‘Transparent Changes’ document (Electronic Supplementary Material [ESM]). Major deviations will also be explicitly stated within the methods section. All data and the code of this research project can be found at GitHub. All analyses were performed using R Version 4.2.0 [31] in the R Studio IDE Version 2022.2.2.485 [32].

### Systematic Scoping Review

The aim of the scoping review was to systematically map current practices of data processing for $\dot{V}{\text{O}}_{2\max }$ determination in the scientific literature. As determining $\dot{V}{\text{O}}_{2\max }$ is a far too common procedure to perform an exhaustive search, we randomly sampled 500 articles published between 2017 and 2022 that referred to $\dot{V}{\text{O}}_{2\max }$ or similar keywords. Data on processing strategies were extracted from all sampled articles that directly measured $\dot{V}{\text{O}}_{2\max }$ using an appropriate testing procedure in human subjects. The review was performed in accordance with the Preferred Reporting Items for Systematic Reviews and Meta-Analyses (PRISMA) Extension for Scoping Reviews [33], see ESM for the checklist.

#### Search and Screening

The article search was conducted on 16 March, 2022 using PubMed and Web of Science. The search included articles published from 2017 to the date of the search referring to ‘maximum oxygen uptake’ or equivalent terms in the title, abstract, or keywords. The ESM shows the exact search terms used.

The search results from both databases were merged and checked for the presence of a Digital Object Identifier. Entries without a Digital Object Identifier were excluded to allow for automated removal of duplicates by Digital Object Identifier matching in the next step. This was followed by an automated title scanning to exclude results that were likely to not be original research articles. All titles that contained one of the following words were excluded: ‘review,’ ‘correction,’ ‘meta-analysis,’ ‘comment,’ ‘retraction,’ ‘editorial,’ ‘erratum,’ ‘reply’.

In accordance with the preregistration, we drew a random sample from the search results. The goal of this process was to give an unbiased estimate of the current state of scientific $\dot{V}{\text{O}}_{2\max }$ testing. The abstracts from the articles included in the random sample were blinded for scanning, by removing any authors identities and journal information. Two of the authors (SN and OJQ) independently scanned the blinded abstracts to filter those that matched one of the exclusion criteria shown in the ESM. When the screeners disagreed in their assessment, they resolved the conflict by discussion.

After the abstract screening, we retrieved the full texts for the remaining articles. The full texts were again independently scanned by two authors (SN and OJQ) to include only those articles that measured $\dot{V}{\text{O}}_{2\max }$ using an appropriate testing procedure in humans (see ESM for the detailed full-text exclusion criteria). Conflicts were resolved by discussion between the two examiners.

#### Data Extraction

We retrieved data from all articles remaining after the abstract and full-text screening. Extraction included the following data:

- metabolic cart used;
- measurement type (breath-by-breath, mixing chamber);
- type of outcome for $\dot{V}{\text{O}}_{2\max }$ (primary, secondary, other);
- data preprocessing (e.g., filtering);
- data processing software;
- interpolation procedure;
- data processing type (time average, breath average, digital filtering, …);
- data processing alignment (moving, binned, …);
- data processing interval (in seconds or breaths, parameters for filtering);
- rationale for the used data processing strategy (e.g., a reference).

The criteria ‘type of outcome’ and ‘rationale’ were added to the extraction list after the abstracts had been scanned, thus they were not stated in the preregistration.

#### Data Synthesis

The extracted data are presented in a purely descriptive way. We calculated the relative and absolute frequency for the reporting of the extracted items. Similarly, we counted the use of different strategies for processing data in all articles that reportedly measured breath-by-breath. The total interval duration of averaging procedures was derived from the reported parameters.

### Experimental Comparison

To determine the influence of the most common data processing strategies on the estimation of $\dot{V}{\text{O}}_{2\max }$, we compared them on a set of already collected gas exchange data from ramp tests in running.

#### Data Source

A total of *N* = 72 exercise tests were analyzed for this study. Because of a miscalculation, the preregistration had incorrectly stated a number of 76 tests. The data were from previous research on the metabolic profile of endurance runners [34, 35]. The tested individuals were experienced distance runners (15 female, 54 male; three of the male individuals participated in both studies). The $\dot{V}{\text{O}}_{2\max }$ tests were conducted in March to September 2019 [34] and March to October 2021 [35], respectively, while using identical exercise protocols and test equipment. Participants ran on a treadmill (saturn 300/100; h/p/cosmos sports & medical 127 GmbH, Nussdorf-Traunstein, Germany) with 1% inclination for 8 min at a velocity of 2.8 m·s^−1^ as a warm-up. After preparing the gas exchange measures, participants started a ramp protocol with an initial speed of 2.8 m·s^−1^ for 2 min and subsequently increased velocity by 0.15 m·s^−1^ every 30 s. The researchers provided verbal encouragement and terminated the exercise when the participants reached subjective exhaustion.

Gas exchange data were recorded using a ZAN 600 USB device (nSpire Health, Inc., Longmont, CO, USA). The device was calibrated with a 3l-syringe pump (nSpire Health, Inc.) and a reference gas (15% O_2_, 6% CO_2_) before each measurement. The measured breath-by-breath data are available on GitHub*.*

#### Data Processing

The spiro package version 0.0.4 for R [36] processed the raw gas exchange data. The software includes various algorithms to calculate $\dot{V}{\text{O}}_{2\max }$ with user-defined parameters. Moving time-based averages were calculated by first linearly interpolating the breath-by-breath data to full seconds. Subsequently, a (center-aligned) moving average was calculated over the specified time span.

For binned time averages, the breath-by-breath data were initially interpolated to full seconds and then binned into consecutive intervals of constant lengths. The average of each interval was aligned to its center. Incomplete intervals (i.e., the last seconds of measurement) were not included in the analysis. Note that some authors use a different procedure for determining their bins, starting by the endpoint of the measurement. However, defining bins beginning at the start of the measurement is a common output option for many gas exchange data analysis software (e.g., Cosmed Omnia). Breath-based moving averages were calculated on the raw data.

#### Comparison of Methods

In response to reviewer comments, we performed statistical analyses not stated in the preregistration. We compared a subset of selected strategies (either strategies suggested by the literature or commonly used in the literature as indicated by our review) using a frequentist mixed model with fixed effects. We investigated the main effect of the strategy and performed corrected post-hoc tests for differences between the processing methods using the R packages lmertest [37] and multcomp [38]. The significance level was set at *α* = 0.05. We used a second more descriptive approach to compare a variety of data processing strategies. The methodology and results of this approach can be seen in the ESM.

## Results

### Systematic Scoping Review

**Fig. 1:**
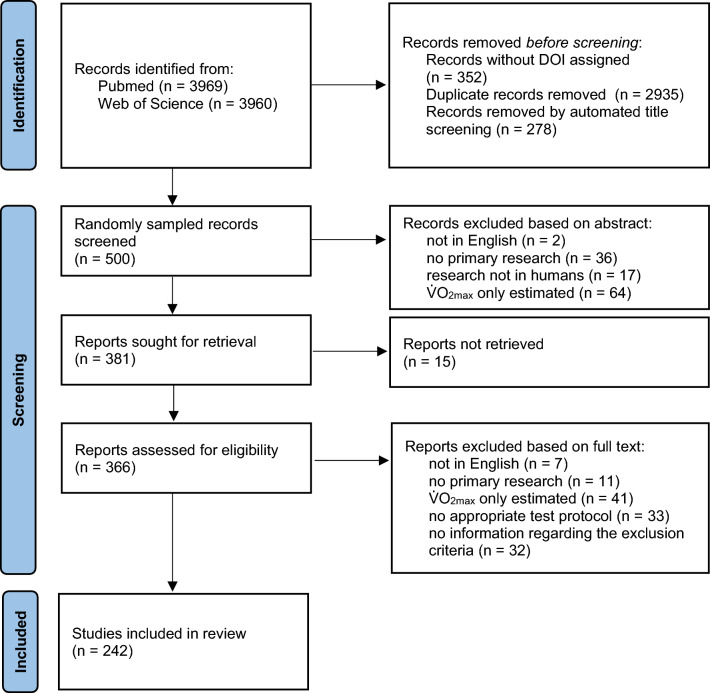
Flow diagram for the systematic scoping review in accordance with the Preferred Reporting Items for Systematic Reviews and Meta-Analyses (PRISMA) 2020 statement [39]. *DOI* Digital Object Identifier, $\dot{V}{\text{O}}_{2\max}$ maximum oxygen uptake

The initial search yielded 7529 results of which 4364 remained after automated filtering and removal of duplicates (see flow diagram in Fig. 1). Out of the random sample (*n* = 500), 242 articles were included in the final analysis.

Reporting practices of the methodology of gas exchange measures differed widely across the literature (see Table 1). Almost half (44.2%) of the articles did not report any information regarding their data processing strategy. About 1 in 20 articles (5.8%) provided a rationale for their used strategy. Only a single article [40] reported information regarding all the investigated criteria.

**Table 1:** Percentage of studies that provided details on the different characteristics of oxygen uptake data processing

Metabolic cart	Preprocessing	Software	Processing strategy	Rationale
88.0%	5.6%^a^	15.0%^a^	55.8%	5.8%

^a^Only examined within the subgroup of studies using breath-by-breath measurements

Out of the studies that provided information and collected breath-by-breath measurements, most (79.5%) utilized binned averages to determine $\dot{V}{\text{O}}_{2\max }$. Moving time averages or breath-based averages were uncommon (see Table 2). No study used digital filtering methods to determine $\dot{V}{\text{O}}_{2\max }$.

**Table 2:** Strategies for processing breath-by-breath data in the reviewed literature (*n* = 88)

Processing strategy	Usage (%)
Binned time	79.5
Moving time	6.8
Moving breath	5.7
Multiple binned time	5.7
Binned breath	2.3

For preprocessing, some authors reported the use of a (linear) interpolation for the breath-by-breath data to seconds (*n* = 7; 4.3%). Few studies reported the use of data filtering strategies to remove outliers. This included the use of initial data smoothing by a short moving average (3 s, *n* = 1; five breaths, *n* = 3), the manual detection and removal of outliers (*n* = 2), or an automated removal of outliers (*n* = 5). For the automated outlier detection, authors removed single data points differing from an unspecified local mean by a varying number of standard deviations (2, 3, or 4) or by being outside of a 95% confidence interval. When reported, the software used for data processing varied among studies showing a total of more than 15 reported programs (for 30 studies that reported this parameter).

The calculation intervals for time-based averages of mixing chamber and breath-by-breath devices ranged from 5 to 60 s (see Table 3). Thirty-second intervals were most common to define $\dot{V}{\text{O}}_{2\max }$, while some authors also often employed shorter (10–20 s) and longer (60 s) periods. For breath-by-breath data, the most common individual data processing strategies were a 30-s binned average (*n* = 30), a 15-s binned average (*n* = 13), a 10-s binned average (*n* = 10), and a 60-s binned average (*n* = 9). The most common strategy not using binned time averages was a 15-breath moving average (*n* = 3).

**Table 3:** Total durations of the calculation interval of maximum oxygen uptake in the reviewed studies

Interval duration (s)	Count
5	1
10	12
15	29
20	12
30	49
40	1
45	1
60	22

### Experimental Comparison

**Fig. 2:**
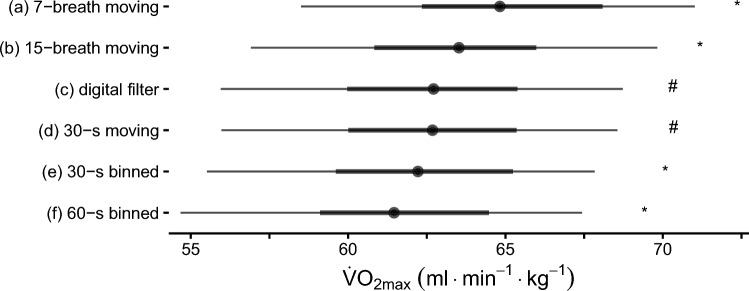
Comparison of selected processing strategies, ordered by their mean relative maximum oxygen uptake ($\dot{V}{\text{O}}_{2\max }$). The black dots and intervals display the mean, 33% (thick line) quantile interval, and 66% (thin line) quantile interval, respectively. There was a significant main effect of strategy on $\dot{V}{\text{O}}_{2\max }$ (*p* < 0.001) with significant differences (*p* ≤ 0.001) between all strategies except the Butterworth filter and the 30-s moving average (*p* = 0.99). **a** Seven-breath moving average, as suggested by Robergs and Burnett [17]. **b** Fifteen-breath moving average, the most common breath-based processing strategy in the reviewed literature. **c** Third-order 0.04-Hz low-pass Butterworth filter, as suggested by Robergs et al. [11]. **d** Thirty-second moving time average, the moving average equivalent to the most common strategy in the reviewed literature. **e** Thirty-second binned time average, the most common data processing strategy in the reviewed literature. **f** Sixty-second binned time average, as suggested by Howley et al. [13]. View the ESM for a similar figure with individual data (S6) and a comparison of more strategies (S5). *min* minutes, *s* seconds, *significantly (*p* < 0.05) different from all other processing strategies; ^#^significantly (*p* < 0.05) different from all other processing strategies apart from the digital filter/30-s moving average

**Fig. 3:**
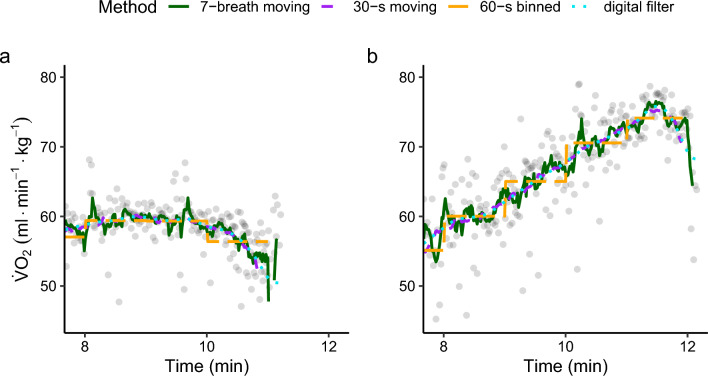
Comparison of data processing strategies for oxygen uptake data from two selected exercise tests. The gray points depict individual breath-by-breath raw data. The displayed digital filter (turquoise, dotted) is a third-order 0.04-Hz low-pass zero-lag (forward–backward) Butterworth filter. **a** An individual displaying a clear plateau with a subsequent decline in oxygen uptake during the final stages of the exercise test. Clearly, data processing strategies with longer calculation intervals yield very similar estimates for $\dot{V}{\text{O}}_{2\max }$, but strategies with short calculation intervals (e.g., seven-breath moving average) may under-process the data and thus overestimate $\dot{V}{\text{O}}_{2\max}$. (**b**) An individual with a constant increase in oxygen uptake without a plateau. Here, processing strategies with longer calculation intervals (e.g., 60-s binned average) may over-process the data and thus underestimate $\dot{V}{\text{O}}_{2\max }$. *min* minutes, *s* seconds

**Fig. 4:**
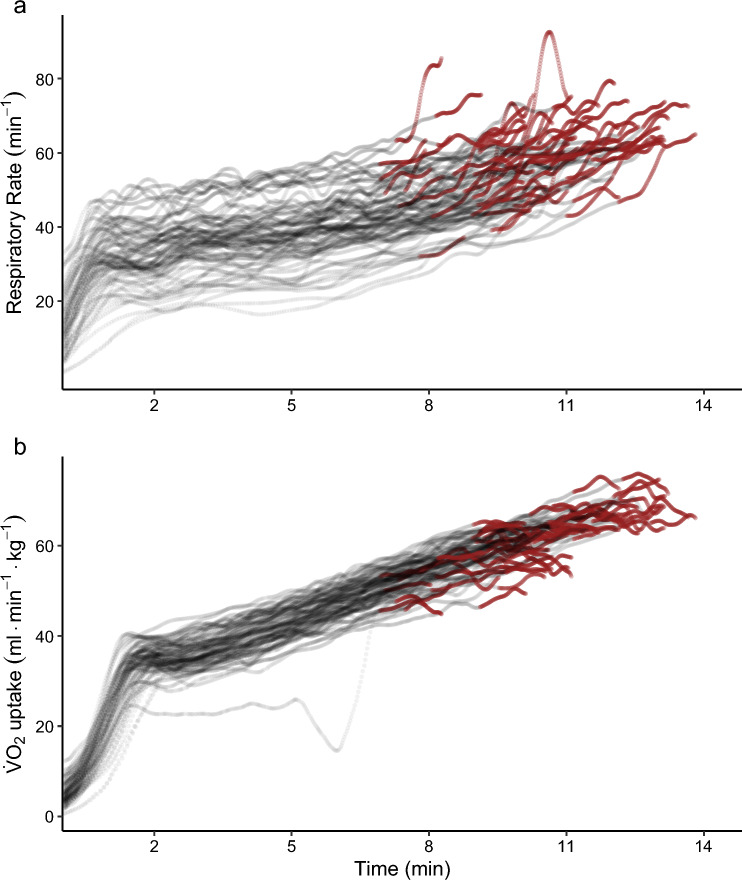
Time courses of respiratory parameters during the ramp tests (*n* = 72). The data were filtered using a 0.04-Hz low-pass third-order Butterworth filter. The red segments correspond to the last minute before exhaustion of each individual. **a** Respiratory rates peak around 60 min^–1^ in the ramp tests. Note that one individual exhibits a sharp spike in the respiratory rate in the minute before exhaustion. **b** Oxygen uptake ($\dot{V}{\text{O}}_{2}$) data show differences in the plateau shape. Note that for one individual, oxygen uptake data were erroneous during the first 7 min of the test

The average $\dot{V}{\text{O}}_{2\max }$ as determined by a binned 30-s average was 62.2 ± 6.3 mL·min^−1^·kg^−1^ (mean ± standard deviation). Applying different data processing strategies for $\dot{V}{\text{O}}_{2\max }$ determination lead to different outcome values (see Fig. 2). The statistical analysis of selected data processing strategies showed a statistically significant main effect for the method chosen (*p* < 0.001, Fig. 2). Post-hoc tests indicated that all strategies differed from each other (*p* < 0.001) with the exception of the digital filter and the 30-s moving average, which showed similar V̇O_2max_ values (*p* = 0.99). On the mean level, the difference between processing strategies can be as high as 5%; on the individual level, they may be much higher (> 10%, see ESM) and vary by individual (see Fig. 3). In general, binned time averages systematically generate lower $\dot{V}{\text{O}}_{2\max }$ values than their moving counterparts (see ESM). When using the same averaging interval, moving time and moving breath averages yield nearly identical values for $\dot{V}{\text{O}}_{2\max }$ in our data set, as most of the trained athletes reached respiratory rates around 60 min^−1^ in the final minutes of the exercise test (see Fig. 4).

## Discussion

We aimed to review current practices of data processing strategies to determine $\dot{V}{\text{O}}_{2\max }$ and to compare them with experimental data. Our results show that recently published research used a wide variety of processing strategies to determine $\dot{V}{\text{O}}_{2\max }$, which directly influences the values obtained. Identical raw breath-by-breath data can result in different $\dot{V}{\text{O}}_{2\max }$ values when processed differently. Moreover, many articles provide only incomplete reports about their chosen methods, which hinders reproducibility of the $\dot{V}{\text{O}}_{2\max }$ measurement.

### Current State of Data Processing

Despite calls to use moving averages or digital filters [11, 17], binned time averages remain the most common data processing strategy to determine $\dot{V}{\text{O}}_{2\max }$ in the reviewed literature (see Table 2). The present findings are generally in agreement with the findings of the non-systematic search by Midgley et al. [22] and the survey by Robergs et al. [11]. It is somewhat surprising that practices have not changed in recent years despite the publication of recommendations discouraging researchers from using binned averages [11]. Using binned time averages leads to systematically lower $\dot{V}{\text{O}}_{2\max }$ values as compared with moving averages (see Fig. 2). The peak in oxygen uptake may be attained between two averaging intervals, resulting in an underestimation of $\dot{V}{\text{O}}_{2\max }$. These errors can be even greater for longer bin widths and when bins cross into early recovery phases or when individuals exhibit rising oxygen profiles without a plateau (see Fig. 3b). As individuals display a wide range of oxygen profiles during the final minutes of the ramp test (see Fig. 4), the magnitude of additional error introduced by binned averages varies by individual. Binned time averages undermine the most important argument in favor of measuring breath-by-breath: the high temporal resolution of data. Despite these arguments speaking against the use of binned time averages, the present review demonstrates that they remain extremely common in the scientific literature.

Breath-based averages seem to be more common (~ 8%) than reported previously (< 1%) [22], but less common than assessed in self-reporting (~ 17%) [11]. The increasing proportion of breath-based averages may be explained by publications in recent years advocating for their use [11, 17]. The length of the calculation interval for averaging is highly diverse within the literature (see Table 3). This may reflect contradictory recommendations [13, 17]. While the optimal calculation interval should depend on the signal characteristics (i.e., the true pulmonary oxygen uptake), the exact features of the physiological signals have not yet been sufficiently researched and can therefore only be modeled theoretically [11]. As different interval durations can influence $\dot{V}{\text{O}}_{2\max }$ by 5% with respect to mean levels (see Fig. 2), the exact reporting of the data processing strategy remains essential for interpretation.

One possible reason for the prevalence of binned average data processing approaches may be due to limitations in the analysis software used. The results show that most researchers use the vendor software of the metabolic cart’s manufacturer. These software packages may by default output binned time averages instead of raw breath-by-breath data. Moreover, further processing (e.g., interpolation, moving averages) may require the use of additional software. This may also explain why digital filtering—despite being recommended by Robergs et al. [11]—has not been used in a single study reviewed here: standard distributions of common data analysis software (e.g., Microsoft Excel) lack the capability to perform such operations. Both more awareness and better software solutions can improve the current practices of data processing.

### Impact of Different Data Processing Strategies

The different data processing strategies found in the literature systematically bias $\dot{V}{\text{O}}_{2\max }$ values (see Fig. 2), and as such influence the classification of individuals, the evaluation of training success, and the assessment of $\dot{V}{\text{O}}_{2\max }$ attainment. In accordance with previous findings [7–9], longer calculation intervals lead to lower $\dot{V}{\text{O}}_{2\max }$ values (see Fig. 2). The analyzed data show mean differences as high as 5% between processing strategies, which is in accordance with previous research [23]. Some studies reported even greater mean differences of up to 20% [10], but only when using raw breath-by-breath data for the comparison. The evaluation of unprocessed raw data for its maximum is highly erroneous and as such is not performed in research (see Table 3); therefore, there is no reason to compare it to other strategies. While previous research was often conducted in sedentary or recreationally trained individuals, the present results provide evidence that a similar effect of data processing strategies on $\dot{V}{\text{O}}_{2\max }$ exists in trained athletes.

As training interventions in trained athletes typically show improvements in $\dot{V}{\text{O}}_{2\max }$ in the range of 0–6% [41, 42], variation caused by differing data processing strategies is approximately of the same magnitude and can bias the evaluation of their success. Together with biological [43] and technical [44] variability, data processing is just one of several sources of variation in the process of $\dot{V}{\text{O}}_{2\max }$ determination, but it is one that can easily be controlled without the need for multiple testing.

Binned time averages lead to systematically lower $\dot{V}{\text{O}}_{2\max }$ values compared with moving averages, for the reasons explained above. While this general trend has been acknowledged previously [20], it has not been quantified. The present data suggest a ~ 1% lower median $\dot{V}{\text{O}}_{2\max }$ when using binned averages compared with moving averages of the same calculation interval length (see ESM). This difference is well within the measurement error of most if not all metabolic carts, but it is systematic and as such may bias the evaluation in scenarios where small changes in $\dot{V}{\text{O}}_{2\max }$ are important (e.g., in high-performance elite sports).

Moving time and moving breath averages with the same averaging interval length lead to almost identical $\dot{V}{\text{O}}_{2\max }$ values with respect to median values (see ESM). This seems natural in that the athletes in this study reached respiratory rates around 60 min^−1^ (see Fig. 4), resulting in equivalent time-based and breath-based interval lengths. For an athletic population, $\dot{V}{\text{O}}_{2\max }$ values obtained by moving time and moving breath averages can approximately be used interchangeably. Given that less trained individuals display lower respiratory rates during exercise tests to exhaustion [45], this finding will likely not generalize to sedentary populations, particularly not to clinical populations with pulmonary diseases.

The exact impact of data processing strategies on the $\dot{V}{\text{O}}_{2\max }$ is highly individual (see ESM). Most research reported only comparisons between average values, with results in accordance with those found here [23]. Data processing strategies may impact $\dot{V}{\text{O}}_{2\max }$ values with varying magnitudes at the individual level. For example, for 10% of the investigated athletes, a binned time average of 5 s leads to a $\dot{V}{\text{O}}_{2\max }$ < 3% greater than by a 30-s average, while for another 10% of the investigated athletes, the $\dot{V}{\text{O}}_{2\max }$ was > 6% greater (see ESM). Current values reported and equations derived compare strategies on a group level [23], which improves the comparison of group results for meta-analyses or group classifications. However, at the individual level, these equations can only be applied with a large margin of error. Differences across data processing strategies on $\dot{V}{\text{O}}_{2\max }$ values range from 1 to 2% in some individuals to more than 10% in others. Hence, when evaluating $\dot{V}{\text{O}}_{2\max }$ data from different tests in a single individual obtained by using different processing methods, there is no way to accurately compare these values even when the processing strategies are reported. While the comparisons of $\dot{V}{\text{O}}_{2\max }$ from different processing strategies require their reporting for a sufficient analysis on a group level, the raw data from each test are required on an individual level.

It is important to note that data processing strategies yielding higher $\dot{V}{\text{O}}_{2\max }$ values are not per se more valid. Short averaging intervals may under-process the data and thus overestimate $\dot{V}{\text{O}}_{2\max }$ (see Fig. 3a). To the contrary, long averaging intervals and binned averages may over-process the “true” signal and thus underestimate $\dot{V}{\text{O}}_{2\max }$ (see Fig. 3b). An adequate processing strategy should find the balance between under-processing and over-processing for a range of different oxygen uptake profiles. In this regard, it is interesting to see that the digital filter recommended by Robergs et al. [11] and the 30-s moving average lead to similar $\dot{V}{\text{O}}_{2\max }$ values in our data set (see Fig. 2, difference filter vs moving average: 0.03 ± 0.27 mL·min^−1^·kg^−1^). This may indicate that a 30-s moving average is an appropriate alternative to the digital filter if the technological requirements to perform the digital filter are not available.

### Guidelines for Reporting

To compare and evaluate $\dot{V}{\text{O}}_{2\max }$ values from different studies, knowledge of the underlying data processing strategies is crucial. Our review demonstrates that almost half of the studies measuring $\dot{V}{\text{O}}_{2\max }$ did not describe their processing strategy. Other aspects of the data processing, such as outlier filtering or the rationale for the chosen procedure, were only in rare instances reported (see Table 1). Table 4 lists seven items that should be reported to provide sufficient information about the data processing strategy used to determine $\dot{V}{\text{O}}_{2\max }$. These items may be reported in the form of a checklist, as an in-text enumeration or in a sentence format. An example paragraph containing all the relevant information for the original data presented in this paper [34, 35] would be:

*“We measured breath-by-breath data during the ramp tests with a ZAN 600 USB device (nSpire Health, Inc., Longmont, CO, United States of America). The unmodified raw data was filtered by using a low-pass forward-backward Butterworth filter (each filter: 3rd order, 0.04 Hz cut-off) implemented in the spiro package for R version 0.0.4 *[36]*. This strategy produces similar results as that recommended by Robergs et al. *[11]*, but does not include a time lag.”*

**Table 4:** Recommendations for reporting data processing strategies to determine the $\dot{V}{\text{O}}_{2\max }$

Reporting item	Description
Metabolic cart	State the exact device model and manufacturer
Measurement mode	State the measurement mode (e.g., mixing chamber, breath-by-breath, …)
Software	State the name and version of the software used for the data analysis
Preprocessing	State if and how data underwent any initial modification (e.g., filtering of outliers, interpolation) before the analysis
Processing strategy	State the exact data processing strategy used to determine the $\dot{V}{\text{O}}_{2\max }$ (e.g., binned time average)
Processing parameters	State the parameters used for the processing strategy (e.g., length of averaging interval)
Rationale	State the rationale for using the processing strategy (e.g., reference to recommendations)

Note that the correct reporting of an exercise test to determine $\dot{V}{\text{O}}_{2\max }$ requires more information than that on data processing. Further aspects to be reported include, but are not limited to: the study population, exercise protocol, device calibration, and criteria to terminate the test. In cases where journals endorse word limits on articles, this reporting—including the reporting on data processing strategies—may be included in supplementary files. The correct and detailed reporting of data processing strategies, as well as other test characteristics, is crucial for interpreting presented $\dot{V}{\text{O}}_{2\max }$ values.

The results of the present work suggest that comprehensive reporting facilitates approximate comparisons of $\dot{V}{\text{O}}_{2\max }$ data on a group level derived using different data processing strategies. However, on an individual level and for a precise comparison, reporting may not be sufficient, as differences between data processing strategies vary between individuals and are potentially influenced by training status. Sharing of the raw gas exchange data can solve this challenge, as it allows researchers to recalculate the $\dot{V}{\text{O}}_{2\max }$ using their preferred data processing strategy. Most raw gas exchange data files are structured in a simple way, which allows the easy removal of any personal information (if this had not been done in the metabolic cart’s system before). In terms of reproducibility of the $\dot{V}{\text{O}}_{2\max }$ determination, sharing anonymized raw data as well as the data analysis code seem to be an even better approach. This requires the data analysis to take place in a programming (or at least a code-generating) environment. Such programs for the purpose of analyzing gas exchange data exist as free open-source software [36, 46].

### Limitations

Because of the sheer number of the publications investigating $\dot{V}{\text{O}}_{2\max }$, it was not possible to perform an exhaustive review of all articles. The scoping review therefore relies on a random sample that may not necessarily capture the exact trends of the literature. However, efforts were made, such as random sampling and systematic article exclusions, to ensure the sample to be representative. Notably, almost half of the studies did not report their data processing strategy at all. The data processing strategies used in the literature could only be investigated when studies reported them.

Ambiguities in the reporting of the investigated studies may impact the analysis results. For example, some studies using long binned averages (e.g., 60 s) may have in fact been using multiple binned averages of a shorter duration (e.g., 4 × 15 s), without describing this correctly. Moreover, the exact definitions for building binned averages vary within the literature. While most studies define the binning periods from the beginning of the exercise, some may define them from the endpoint. We performed a 1-s interpolation prior to the calculation of the binned averages, a procedure that seems reasonable from a data processing viewpoint but was only reported in a few instances in the reviewed literature. While these two variants of binned averages (period definition from the end of the exercise and no prior interpolation) can lead to a different $\dot{V}{\text{O}}_{2\max }$ on the individual level, they did not yield to any meaningful differences in $\dot{V}{\text{O}}_{2\max }$ on the group level in our data set when compared to the 30-s binned average as defined in our methods (0.05 ± 0.48 and 0.09 ± 0.26 mL·min^−1^·kg^−1^). Additionally, some of the included studies did not define the maximum bin, but a pre-set binned average period as their $\dot{V}{\text{O}}_{2\max }$ (e.g., the last bin, regardless of its value). In situations where the maximum in oxygen uptake is reached considerably before exhaustion (i.e., a long plateau in oxygen uptake exists), this may lead to different results than a traditional binned average processing. We did not separately consider such sub-categories of data processing strategies, as they may not be very common and are often hard to investigate precisely because of ambiguities in their reporting.

This work treated each breath as the single data processing unit of cardiopulmonary exercise testing. However, metabolic carts sample gas fraction and gas flow data at a much greater frequency (e.g., 50 Hz). Subsequently, the data for each breath is calculated from the raw signals. Different algorithms to generate the breath-by-breath data can lead to different outcomes [47], and accordingly may also influence $\dot{V}{\text{O}}_{2\max }$ estimates. Hence, documenting and reporting of the breath-by-breath algorithm seem warranted. Yet, many metabolic carts do not describe their default algorithm and limit access to the raw data signal.

The experimental comparison of different data processing strategies was conducted on a standardized data set of exercise tests. This standardization in terms of training status, exercise protocol, and measurement device helps to highlight the impact of different data processing strategies even in a relatively homogeneous data set. However, the results may only partly transfer to different settings, such as less fit individuals.

## Conclusions

Despite calls for standardization, current research uses a variety of data processing strategies to determine the $\dot{V}{\text{O}}_{2\max }$ from raw gas exchange data. The by far most common strategy, a 30-s binned average, systematically underestimates “true” $\dot{V}{\text{O}}_{2\max }$ and thus should be avoided. While digital filtering remains the most reasonable approach to process oxygen uptake data, a 30-s moving average may sufficiently approximate its results in a trained population. Based on current reporting practices, we developed a checklist that can serve as a guideline for reporting data processing methods for $\dot{V}{\text{O}}_{2\max }$ determination. Based on the current findings, authors should follow reporting guidelines and ideally share anonymized raw data to improve the reproducibility of research in exercise physiology.

### Supplementary Information

Below is the link to the electronic supplementary material.

Supplementary file1 (PDF 554 KB)

## Declarations

### Availability of Data and Material

All data generated for this study are available at https://github.com/smnnlt/vo2max-processing.

### Code Availability

The code generated for this study is available at https://github.com/smnnlt/vo2max-processing.
