# Supplementary material for: Data Processing Strategies to Determine Maximum Oxygen Uptake: A Systematic Scoping Review and Experimental Comparison with Guidelines for Reporting
Source: Sports Med. 2023 Aug 21;53(12):2463–75. doi: 10.1007/s40279-023-01903-3 (PMC10687136; doi:10.1007/s40279-023-01903-3)
Supplement: Supplementary file 1 — Supplementary file1 (PDF 554 KB) [file 40279_2023_1903_MOESM1_ESM.pdf]

Data Processing Strategies to Determine Maximum Oxygen Uptake: A Systematic Scoping Review and Experimental Comparison with Guidelines for Reporting

Supplementary Information

Simon Nolte[1*], Robert Rein[2], Oliver Jan Quittmann[1]

[1] Institute of Movement and Neurosciences, German Sport University Cologne, Germany

[2] Institute of Exercise Training and Sport Informatics, German Sport University Cologne, Germany

* Correspondence: s.nolte@dshs-koeln.de

Note:

All data and code for the manuscript is publicly available at https://github.com/smnnlt/vo2max-processing.

The preregistration is available at https://osf.io/3am4s.

S1: Transparent Changes 2
S2: PRISMA-ScR Checklist 4
S3: Table: Search Terms 6
S4: Table: Exclusion Criteria 6
S5: Plot: Descriptive comparison of several processing strategies 7
S6: Plot: Comparison of selected processing strategies with individual data 8
References 9

S1: Transparent Changes

This supplementary document includes all deviations and modifications of code and methods in the final project compared to its preregistration.

Major Changes

Number of exercise tests for comparison

Due to a miscalculation, the preregistration provided an incorrect number of exercise test (n = 76). The correct number of exercise tests is n = 72, with 44 from Quittmann et al. (2022) (one test only partly included in the original work due to missing other data) and 28 from Schwarz et al. (2022) (three tests only partly included in the original work due to missing other data).

Additional variables extracted from included research

`outcome`: Which type of outcome $\dot{V}O_{2max}$ is. Either `primary`, `secondary` or `other`

`source`: Which source is provided for the data processing method used.

Additional statistical analysis

In response to reviewer feedback we added additional statistical analyses to compare selected data processing strategies.

Minor Changes

Code changes for automated article filtering and screening preparation

- advanced detection of missing DOIs: `is.na(merge_data$doi) | (merge_data$doi == "")` instead of `is.na(merge_data$doi)`.
- Improved function to retrieve missing PubMed abstracts: Handles case when input (PMID) is missing (`if (is.na(pmid)) return(NA)).`
- save/load of the sampling results as an .Rda file to reduce computation time when working on parts of the workflow.

Unblinding of single abstracts

- Manual retrieval of abstracts for articles, as these were neither present in the search result data, nor could be automatically scraped. Abstracts were saved and imported as .txt files. Temporary unblinding only applied to the primary researcher. This concerns the abstracts with the sampling id (sid): 50, 238, 288, 416, 488, 490, 500
- Manual retrieval of abstracts for articles, as the automatically collected abstract contained html-tags that could not be removed for later abstract plots. Abstracts were saved and imported as .txt files. Temporary unblinding only applied to the primary researcher. This concerns the abstracts with the sampling id (sid): 344, 356
- Unblinding during screening to assess the implications of title given in squared brackets. This concerns the abstracts with the sampling id (sid): 262, 303
- Consulting of online abstract due to incomplete abstract plot. This concerns the abstract with the sampling id (sid): 275

Minor Modification of exclusion criteria

Changes are in italics:

`'r'`: Is the article no original research *(i.e. no primary analysis of experimental data*) ? (if yes, indicate 'r'; if no, continue)

`'t'`: Was no full-text ~~available~~ *accessible* for the corresponding article? (if yes, indicate 't', if no continue)

Minor screening error for two articles

For two articles (sampling ids: 194, 282) I only realized during data extraction that they matched the exclusion criteria (`c`: no continuous measurement of oxygen uptake). In agreement of both screeners, the screening data was retrospectively changed.

S2: PRISMA-ScR Checklist

Checklist taken from Tricco et al. (2018)

Preferred Reporting Items for Systematic reviews and Meta-Analyses extension for Scoping Reviews (PRISMA-ScR) Checklist

SECTION	ITEM	PRISMA-ScR CHECKLIST ITEM	REPORTED IN SECTION
**TITLE**			
Title	1	Identify the report as a scoping review.	Title
**ABSTRACT**			
Structured summary	2	Provide a structured summary that includes (as applicable): background, objectives, eligibility criteria, sources of evidence, charting methods, results, and conclusions that relate to the review questions and objectives.	Abstract
**INTRODUCTION**			
Rationale	3	Describe the rationale for the review in the context of what is already known. Explain why the review questions/objectives lend themselves to a scoping review approach.	1 Introduction, paragraph 6
Objectives	4	Provide an explicit statement of the questions and objectives being addressed with reference to their key elements (e.g., population or participants, concepts, and context) or other relevant key elements used to conceptualize the review questions and/or objectives.	1 Introduction, paragraph 6; 2.1 Systematic Scoping Review
**METHODS**			
Protocol and registration	5	Indicate whether a review protocol exists; state if and where it can be accessed (e.g., a Web address); and if available, provide registration information, including the registration number.	2 Methods
Eligibility criteria	6	Specify characteristics of the sources of evidence used as eligibility criteria (e.g., years considered, language, and publication status), and provide a rationale.	2.1.1 Search & Screening
Information sources*	7	Describe all information sources in the search (e.g., databases with dates of coverage and contact with authors to identify additional sources), as well as the date the most recent search was executed.	2.1.1 Search & Screening
Search	8	Present the full electronic search strategy for at least 1 database, including any limits used, such that it could be repeated.	S3
Selection of sources of evidence†	9	State the process for selecting sources of evidence (i.e., screening and eligibility) included in the scoping review.	2.1.1 Search & Screening; S4
Data charting process‡	10	Describe the methods of charting data from the included sources of evidence (e.g., calibrated forms or forms that have been tested by the team before their use, and whether data charting was done independently or in duplicate) and any processes for obtaining and confirming data from investigators.	2.1.2 Data Extraction
Data items	11	List and define all variables for which data were sought and any assumptions and simplifications made.	2.1.2 Data Extraction

SECTION	ITEM	PRISMA-ScR CHECKLIST ITEM	REPORTED IN SECTION
Critical appraisal of individual sources of evidence§	12	If done, provide a rationale for conducting a critical appraisal of included sources of evidence; describe the methods used and how this information was used in any data synthesis (if appropriate).	N/A
Synthesis of results	13	Describe the methods of handling and summarizing the data that were charted.	2.1.3 Data Synthesis
**RESULTS**			
Selection of sources of evidence	14	Give numbers of sources of evidence screened, assessed for eligibility, and included in the review, with reasons for exclusions at each stage, ideally using a flow diagram.	Figure 1
Characteristics of sources of evidence	15	For each source of evidence, present characteristics for which data were charted and provide the citations.	Online document
Critical appraisal within sources of evidence	16	If done, present data on critical appraisal of included sources of evidence (see item 12).	N/A
Results of individual sources of evidence	17	For each included source of evidence, present the relevant data that were charted that relate to the review questions and objectives.	3.1 Systematic Scoping Review; online document
Synthesis of results	18	Summarize and/or present the charting results as they relate to the review questions and objectives.	3.1 Systematic Scoping Review; Table 1-3
**DISCUSSION**			
Summary of evidence	19	Summarize the main results (including an overview of concepts, themes, and types of evidence available), link to the review questions and objectives, and consider the relevance to key groups.	4.1 Current State of Data Processing
Limitations	20	Discuss the limitations of the scoping review process.	4.4 Limitations
Conclusions	21	Provide a general interpretation of the results with respect to the review questions and objectives, as well as potential implications and/or next steps.	4.1 Current State of Data Processing 4.3 Guidelines for Reporting
**FUNDING**			
Funding	22	Describe sources of funding for the included sources of evidence, as well as sources of funding for the scoping review. Describe the role of the funders of the scoping review.	Funding Statement

JBI = Joanna Briggs Institute; PRISMA-ScR = Preferred Reporting Items for Systematic reviews and Meta-Analyses extension for Scoping Reviews.
* Where *sources of evidence* (see second footnote) are compiled from, such as bibliographic databases, social media platforms, and Web sites.
† A more inclusive/heterogeneous term used to account for the different types of evidence or data sources (e.g., quantitative and/or qualitative research, expert opinion, and policy documents) that may be eligible in a scoping review as opposed to only studies. This is not to be confused with *information sources* (see first footnote).
‡ The frameworks by Arksey and O'Malley (6) and Levac and colleagues (7) and the JBI guidance (4, 5) refer to the process of data extraction in a scoping review as data charting.
§ The process of systematically examining research evidence to assess its validity, results, and relevance before using it to inform a decision. This term is used for items 12 and 19 instead of "risk of bias" (which is more applicable to systematic reviews of interventions) to include and acknowledge the various sources of evidence that may be used in a scoping review (e.g., quantitative and/or qualitative research, expert opinion, and policy document).

S3: Table: Search Terms

Table S3: Search strings for the systematic scoping review.

Source	Search Strings
PubMed	‘(((((”maximum oxygen uptake”) OR (”maximal oxygen uptake”)) OR (”VO2max”)) OR (”maximum oxygen consumption”)) OR (”maximal oxygen consumption”)) AND ((”2017/01/01”[Date - Publication] : ”3000”[Date - Publication]))‘
Web of Science	‘(((((ALL=(”maximum oxygen uptake”)) OR ALL=(”maximal oxygen uptake”)) OR ALL=(”VO2max”)) OR ALL=(”maximum oxygen consumption”)) OR ALL=(”maximal oxygen consumption”)) AND PY=(2017-2022)‘

S4: Table: Exclusion Criteria

Table S4: Exclusion criteria for the screening process.

	Criterion	Details
A*	not in English	full text only available in non-English language
B*	no primary research	research was no original investigation or only a reanalysis of data
C*	Research not in humans	research was conducted in animals
D*	$\dot{V}O_{2max}$ only estimated	$\dot{V}O_{2max}$ was only approximated by means of a predictive equation
E	No appropriate testing protocol	protocol for $\dot{V}O_{2max}$ testing did either not include exercise to voluntary exhaustion or was to long (>20 min) for a reliable estimate
F	No information regarding the exclusion criteria	crucial information on $\dot{V}O_{2max}$ testing that allowed the evaluation of the other exclusion criteria were missing

*During the abstract screening only the criteria marked with an asterix were evaluated

S5: Plot: Descriptive comparison of several processing strategies

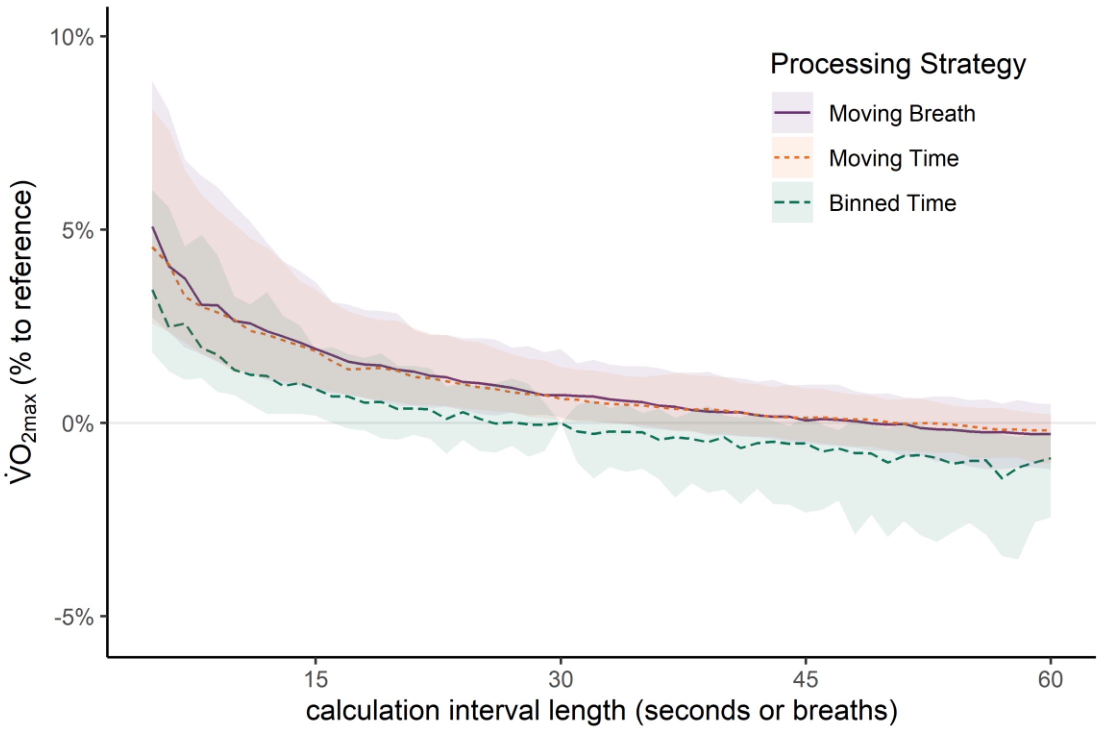

***Fig. S5**: V̇O_{2max} varies by data processing strategy. Values are expressed relative to the V̇O_{2max} from a 30-second binned average — the most common strategy as determined by the review. The lines display the median, the shaded areas mark the interval between 10th and 90th percentile. Using moving average leads to systematically higher V̇O_{2max} values compared to binned time averages. Changing the averaging interval (in seconds or breaths) can lead to median changes in V̇O_{2max} as large as 5%.*

Methodology:

For our descriptive comparison of a variety of processing strategies we chose to express the V̇O_{2max} normalized to a reference procedure. The reference procedure was the one most commonly applied in the current literature as determined by our scoping review (30-second binned average). Individual V̇O_{2max} values were expressed in reference to this procedure, where a value of 1 means that the processing method yields exactly the same V̇O_{2max} value as the reference method. We calculated the data for all integer parameter values within the range of the calculation intervals found in the literature during the review (5-60) for the three strategies of moving breath averages, moving time averages, and binned time averages. At the group level, we calculated the median and 10%- and 90%-quantiles of each processing strategy.

S6: Plot: Comparison of selected processing strategies with individual data

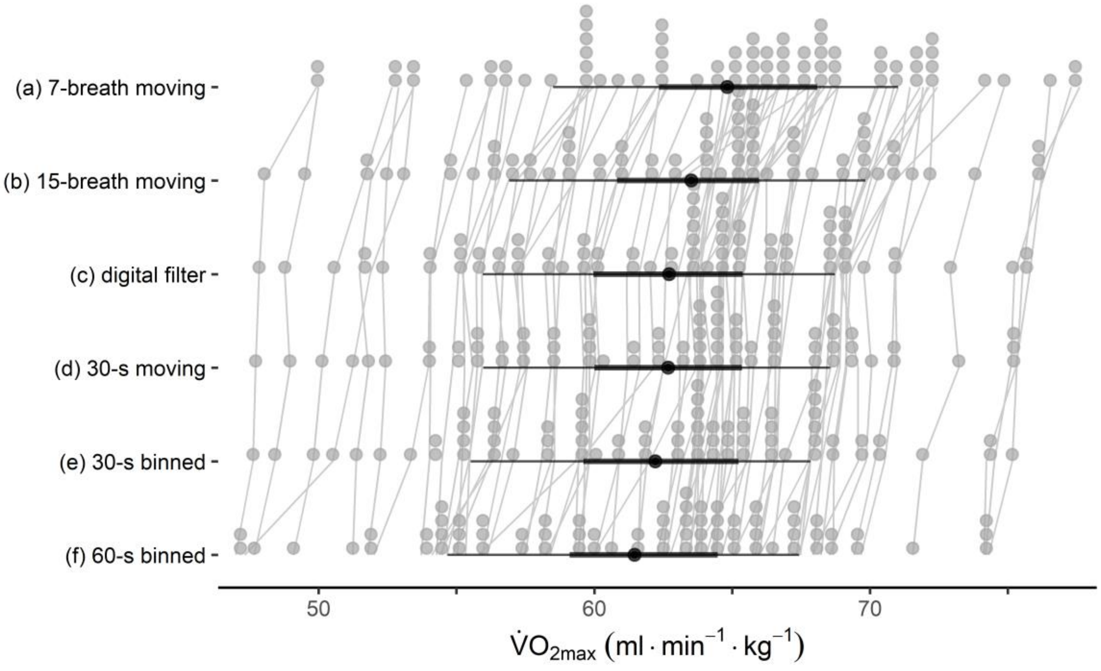

Fig. S6*: Comparison of selected processing strategies, ordered by their mean relative $\dot{V}O_{2max}$, including individual data. The black dots and intervals display the mean and 33%/66% quantile intervals, respectively. Gray points and lines display the individual data points. There was a significant main effect of strategy on $\dot{V}O_{2max}$ ($p < 0.001$) with significant differences ($p \leq 0.001$) between all strategies except the Butterworth filter and the 30-second moving average ($p = 0.99$). (a) 7-breath moving average, as suggested by Robergs & Burnett (2003). (b) 15-breath moving average, the most common breath-based processing strategy in the reviewed literature. (c) A third order 0.04 Hz low-pass Butterworth filter, as suggested by Robergs et al. (2010). d) 30-second moving time average, the moving average equivalent to the most common strategy in the reviewed literature. (e) 30-second binned time average, the most common data processing strategy in the reviewed literature. (f) 60-second binned time average, as suggested by Howley et al. (1995).*

References

1.

Howley ET, Bassett DR, Welch HG. Criteria for maximal oxygen uptake: review and commentary. Medicine & Science in Sports & Exercise. 1995;27:1292–301. https://doi.org/10.1249/00005768-199509000-00009

Quittmann OJ, Foitschik T, Vafa R, Freitag F, Spearmann N, Nolte S, et al. Is maximal lactate accumulation rate promising for improving 5000-m prediction in running? International Journal of Sports Medicine. 2022; https://doi.org/10.1055/a-1958-3876

Robergs RA, Burnett A. Methods used to process data from indirect calorimetry and their application to VO2max. Journal of Exercise Physiology Online. American Society of Exercise Physiologists; 2003;6:44–57.

Robergs RA, Dwyer D, Astorino T. Recommendations for Improved Data Processing from Expired Gas Analysis Indirect Calorimetry. Sports Med. 2010;40:95–111. https://doi.org/10.2165/11319670-000000000-00000

Schwarz YM, Nolte S, Fuchs M, Gehlert G, Slowig Y, Schiffer A, et al. Relationship between physiological parameters and time trial performance over 1, 2 and 3 km in well-trained runners. 27th Annual Congress of the European College of Sport Science: Book of Abstracts. ECSS; 2022. p. 308–8.

Tricco AC, Lillie E, Zarin W, O'Brien KK, Colquhoun H, Levac D, et al. PRISMA extension for scoping reviews (PRISMA-ScR): Checklist and explanation. Annals of Internal Medicine. 2018;169:467–73. https://doi.org/10.7326/M18-0850
